# Targeted Genome Replacement via Homology-directed Repair in Non-dividing Cardiomyocytes

**DOI:** 10.1038/s41598-017-09716-x

**Published:** 2017-08-24

**Authors:** Takamaru Ishizu, Shuichiro Higo, Yuki Masumura, Yasuaki Kohama, Mikio Shiba, Tomoaki Higo, Masato Shibamoto, Akito Nakagawa, Sachio Morimoto, Seiji Takashima, Shungo Hikoso, Yasushi Sakata

**Affiliations:** 1 Department of Cardiovascular Medicine, Osaka University Graduate School of Medicine, Suita, Osaka, 565-0871 Japan; 2 Department of Health and Medical Care, International University of Health and Welfare, Okawa, Fukuoka, 831-8501 Japan; 3 Department of Medical Biochemistry, Osaka University Graduate School of Medicine, Suita, Osaka, 565-0871 Japan; 4 Department of Medical Therapeutics for Heart Failure, Osaka University Graduate School of Medicine, Suita, Osaka, 565-0871 Japan

## Abstract

Although high-throughput sequencing can elucidate the genetic basis of hereditary cardiomyopathy, direct interventions targeting pathological mutations have not been established. Furthermore, it remains uncertain whether homology-directed repair (HDR) is effective in non-dividing cardiomyocytes. Here, we demonstrate that HDR-mediated genome editing using CRISPR/Cas9 is effective in non-dividing cardiomyocytes. Transduction of adeno-associated virus (AAV) containing sgRNA and repair template into cardiomyocytes constitutively expressing Cas9 efficiently introduced a fluorescent protein to the C-terminus of *Myl2*. Imaging-based sequential evaluation of endogenously tagged protein revealed that HDR occurs in cardiomyocytes, independently of DNA synthesis. We sought to repair a pathological mutation in *Tnnt2* in cardiomyocytes of cardiomyopathy model mice. An sgRNA that avoided the mutated exon minimized deleterious effects on Tnnt2 expression, and AAV-mediated HDR achieved precise genome correction at a frequency of ~12.5%. Thus, targeted genome replacement via HDR is effective in non-dividing cardiomyocytes, and represents a potential therapeutic tool for targeting intractable cardiomyopathy.

## Introduction

Over the past few decades, the development of medical therapies has reduced mortality in patients with heart failure; however, the prognosis of patients with advanced heart failure caused by idiopathic cardiomyopathy still remains poor even under the most intensive pharmacological and non-pharmacological therapies^1, 2^. Genetic abnormalities are widely recognized as a major etiological basis of cardiomyopathy, and recent advances in high-throughput sequencing technologies have revealed the high incidence of pathological genomic mutations in both familial and sporadic cardiomyopathies^3–5^. The precise repair of a mutation in a causative gene has the potential for radical preventive therapy against the development of heart failure caused by upstream genetic defects. To date, however, the genomic mutation themselves have not been recognized as direct targets for therapeutic intervention. CRISPR/Cas9 genome editing technologies are increasingly recognized as potential tools for directly correcting genetic mutations in diseased cells and tissues^6^. Genome editing therapies using programmable nucleases, combined with designed repair template DNA, have been rapidly developed to treat intractable disease such as viral infection^7^, enzymatic deficiency^8^, and hereditary myopathies^9–11^. Genomic cleavages after DNA double-strand breaks (DSB) are repaired through non-homologous end-joining (NHEJ) or homology-directed repair (HDR) pathways^12–17^. In contrast to error-prone NHEJ, which results in the formation of an insertion or deletion at the DSB site, HDR enables accurate genome repair using exogenously introduced single- or double-stranded DNA templates. However, HDR occurs primarily during S/G2 phase, and is thus restricted to cells that are actively dividing^6, 16, 18–22^, limiting its application in non-dividing cells such as cardiomyocytes.

Here, we introduced genome-editing components, including HDR template, into cultured cardiomyocytes constitutively expressing Cas9, and then evaluated genome editing over a time course using an imaging cytometer. Sequential observation of individual cells expressing endogenously tagged fluorescent protein fused to cardiac specific myosin regulatory light chain (Myl2) gene revealed that HDR occurred in non-dividing cardiomyocytes that did not enter S phase. Furthermore, we sought to repair a pathological deletion mutation in the *Tnnt2* gene in cardiomyocytes in dilated cardiomyopathy (DCM) model mice, and achieved precise genome correction at a rate of ~12.5%.

## Results

### Establishment of an evaluation method to detect HDR using a high-content image cytometry

**Figure 1:**
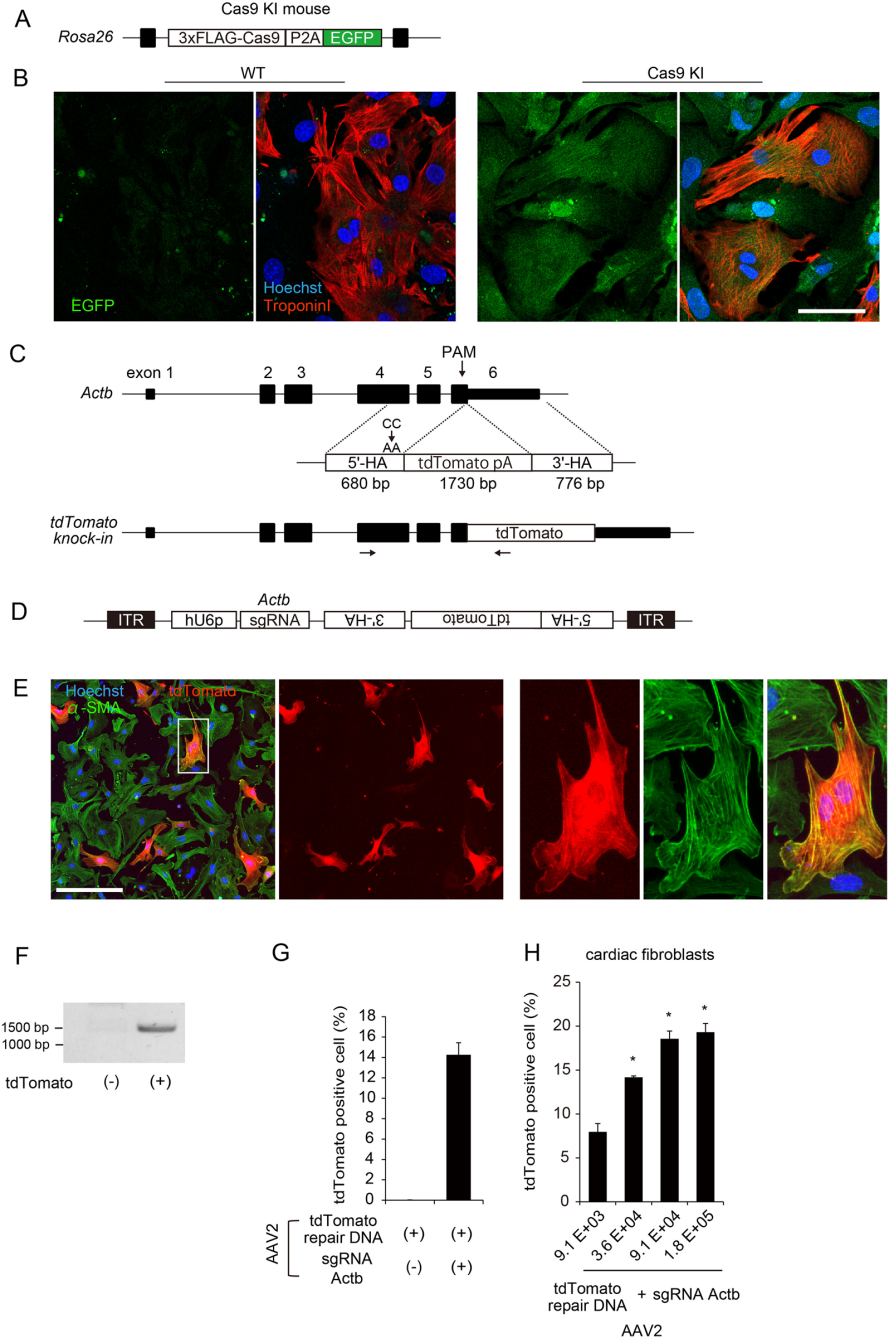
Establishment of an evaluation method to detect HDR using a high-content image cytometry. (**A**) Cas9 knock-in mouse harbors a transgene encoding FLAG-Cas9-P2A-EGFP fusion protein at the *Rosa26* locus. (**B**) Isolated cardiomyocytes and non-cardiomyocytes were cultured, fixed, and immunostained with anti–Troponin I antibody. EGFP signals were observed in both cell types. Scale bar: 50 μm. (**C**) The mouse *Actb* consists of 6 exons (upper). In the HDR repair template, the 1730-bp tdTomato fluorescent protein and 3′-terminal poly A sequence was cloned in-frame between the 680-bp 5′-terminal and 776-bp 3′-terminal homology arms (5′-HA and 3′-HA, respectively) corresponding to the genomic sequence of the 3′-terminus of *Actb*. PAM mutation (CC to AA) was introduced into 5′-HA, and stop codon sequence was removed. Expected genomic sequence of Actb-tdTomato fusion gene after successful HDR (lower). Arrows indicate locations of PCR primers (forward primer was designed outside homology arm). (**D**) Design of AAV vector. hU6 promoter (hU6p)-driven sgRNA and tdTomato HDR template including 5′-terminal homology arm (5′-HA) and 3′-terminal homology arm (3′-HA) were subcloned between inverse terminal repeat (ITR) sequences. (**E**) Cardiac fibroblasts isolated from Cas9 knock-in mice were seeded in 96-well plates and transduced with AAV serotype 2 (AAV2) encoding sgRNA and HDR template. Forty-eight hours after transduction, cells were fixed and stained with anti–α-SMA antibody. tdTomato-positive cell in white square is enlarged in right panels. Scale bar: 100 μm. (**F**) Cardiac fibroblasts were transduced with AAV as in (**G**). Forty-eight hours after transduction, genomic DNA were extracted from tdTomato-positive or –negative fibroblasts sorted by FACS. Genomic PCR was performed using a primer pair targeting inside and outside the homology arm (Fig. 1C). (**H**) Cardiac fibroblasts seeded in 96-well plates were transduced using AAV2 encoding HDR template with or without sgRNA targeting *Actb*. Forty-eight hours after transduction, cells were fixed and immunostained. The proportion of tdTomato-positive fibroblasts (among all cells) was calculated using the image cytometry (n = 3, means ± SD). (**I**) Cardiac fibroblasts seeded in 96-well plates were transduced using purified AAV in increasing titers (viral genomes per cell). Forth-eight hours after transduction, the proportion of tdTomato-positive fibroblasts were calculated as in (H). n = 3, *p < 0.01 vs low titer (9.1E + 03).

One of the greatest challenges related to achieving HDR in primary cultured cells such as cardiomyocytes is the introduction of the large Cas9 protein. Therefore, we used cells isolated from hearts of genetically modified Cas9 knock-in mice in which 3 × FLAG-fused Cas9 and a P2A self-cleavable peptide followed by EGFP protein are knocked in at the endogenous *Rosa26* locus^23^ (Fig. 1A). The Cas9 knock-in mouse was crossed with a β-actin Cre driver mouse, resulting in ubiquitous expression of Cas9-P2A-EGFP in all tissues^23^, including cardiomyocytes and non-cardiomyocytes (Fig. 1B). We first sought to establish an imaging-based evaluation method for detecting successful HDR in primary cultured dividing cells using a high-content image cytometry (IN Cell Analyzer 6000). We targeted the mouse *Actb* gene, which encodes β-actin, a structural protein ubiquitously expressed in cells and tissues. Four candidate single guide RNAs (sgRNAs) targeting the genomic region around the stop codon of *Actb* were selected using a CRISPR design tool^24^. Cleavage activity was evaluated using single-strand annealing^25^ and mismatch-specific nuclease assays (Fig. S1A and B), and sgRNA #2, which targeted the PAM sequence just upstream of the stop codon of *Actb*, had the most efficient cleaving activity. We generated an HDR template vector harboring the 1730-bp coding sequence of tdTomato fluorescent protein followed by a polyadenylation signal between the homology arms of the 3′-terminal region of *Actb* to detect fluorescent signals produced from the fusion protein expressed from the endogenous locus (Fig. 1C and Fig. S1C). To transduce primary cultured cardiac cells, we used an adeno-associated virus (AAV) encoding a human U6 promoter–driven sgRNA targeting mouse *Actb* and the HDR template sequence between AAV inverted terminal repeat (ITR) sequences (Fig. 1D). We then isolated non-cardiomyocytes from neonatal mouse hearts, most of which are proliferative cardiac fibroblasts^26^ positive for α-smooth muscle actin (α-SMA) or vimentin (Fig. S1D). Immunostaining with anti α-SMA antibody and Alexa Fluor 488–conjugated secondary antibody clearly detected endogenous actin filaments even in the presence of the background EGFP signal generated by Cas9-P2A-EGFP (Fig. S1D). Cardiac fibroblasts isolated from Cas9 knock-in mice were seeded in 96-well plates and transduced with AAV serotype 2 (AAV2) encoding the sgRNA and HDR template. Forty-eight hours after transduction, the cells were fixed and stained with anti α-SMA antibody. As shown in Fig. 1E, fibroblasts positive for α-SMA and tdTomato fluorescent signals were observed 48 h after transduction. The tdTomato fluorescent signal colocalized with α-SMA protein (Fig. 1E, right panels), suggesting that the Actb-tdTomato fusion protein precisely localized in cytoskeletal structures of cardiac fibroblasts. Genomic PCR using primers targeting outside and inside the homology arms, followed by Sanger sequencing, confirmed precise genomic recombination of the HDR template at the *Actb* locus (Fig. 1F and Fig. S1E). Transduction of AAV2 encoding the HDR template but lacking the sgRNA targeting *Actb* did not yield tdTomato-positive cardiac fibroblasts (Fig. 1G). Transduction of purified AAV2 into cardiac fibroblasts improved HDR efficiency in a dose-dependent manner, reaching a plateau at approximately 20% (Fig. 1H). In western blots, Actb-tdTomato fusion protein was detected at the expected molecular weight (Fig. S1F). The molar ratio of Actb-tdTomato fusion protein estimated from western blot was 2.0%, suggesting that Actb-tdTomato fusion protein might be conformationally unstable and easily degraded. We sequentially observed cardiac fibroblasts transduced with AAV2 and found that tdTomato-positive fibroblasts gradually increased until day 6 (Fig. S1G). Cytoskeletal structures in these cells (intermediate filament detected by Vimentin staining and actin filament detected by α-SMA staining) were not significantly affected by tagging of tdTomato fluorescent protein to Actb (Fig. S1H). These results suggest that a high-content image cytometry is a useful tool to detect HDR, enabling estimation of HDR per cells treated with the genome editing components in primary cultured cardiac fibroblasts.

### Elucidation of the time course of HDR by sequential observation of individual cardiomyocytes

**Figure 2:**
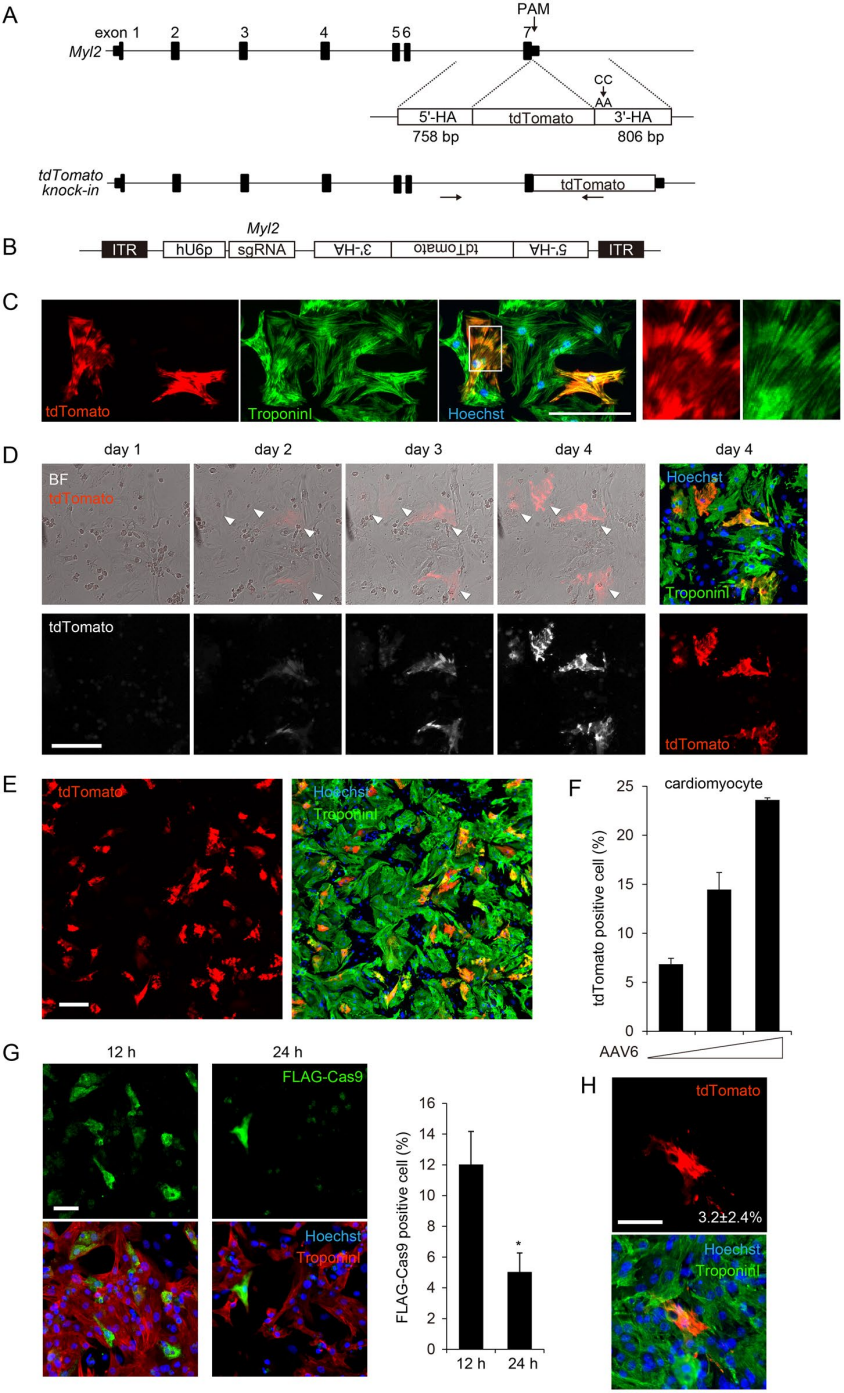
Elucidation of the time course of HDR by sequential observation of individual cardiomyocytes. (**A**) The mouse *Myl2* gene locus consists of 7 exons. HDR repair template consists of coding sequence of tdTomato fluorescent reporter protein between 758-bp 5′-terminal and 806-bp 3′-terminal homology arms (5′-HA and 3′-HA) corresponding to the genomic sequence of the 3′-terminus of *Myl2*. PAM mutation (CC to AA) was introduced into 3′-HA, and the stop codon sequence was removed. Arrows indicate the locations of PCR primers (outer primer was designed outside the homology arm). (**B**) Design of AAV vector. hU6 promoter (hU6p)-driven sgRNA and tdTomato HDR template were subcloned between inverse terminal repeat (ITR) sequences. (**C**) Cardiomyocytes isolated from Cas9 knock-in mice were seeded in 96-well plates and transduced with AAV serotype 6 (AAV6) encoding sgRNA and HDR template. Forty-eight hours after transduction, cells were fixed and stained with anti-troponin antibody. tdTomato-positive cardiomyocytes in white square are enlarged in right panels. Scale bar: 100 μm. (**D**) Cardiomyocytes were treated as in (**C**). After transduction of AAV, bright-field and fluorescence images were sequentially obtained using the image cytometry targeting the same fields determined by coordinate axes. On day 4, cells were fixed and immunostained. Arrowheads indicate the tdTomato-positive cardiomyocytes. Scale bar: 100 μm. (**E**) Immunostaining image of cardiomyocytes transduced with purified AAV6 encoding HDR components. Scale bar: 100 μm. (**F**) Cardiomyocytes were transduced with AAV6 at increasing viral titer (2.17 × 10^4^, 4.34 × 10^4^, and 1.09 × 10^5^ viral genomes/cell). At day 4, cells were fixed and immunostained. The proportion of tdTomato-positive cells among troponin I–positive cardiomyocytes was calculated using the image cytometry (n = 3, means ± SD). (**G**) Cardiomyocytes isolated from neonatal WT mice were transfected with mRNA encoding N-terminally FLAG-tagged Cas9. Twelve and 24 h after transfection, cells were fixed and immunostained. Scale bar: 50 μm. The proportion of FLAG-Cas9-positive cells among troponin I–positive cardiomyocytes was calculated using the image cytometry (n = 3, means ± SD, *: p < 0.05 vs 12 h). (**H**) WT cardiomyocytes were transduced with AAV6 (4.34 × 10^4^ viral genomes/cell). Twelve h after transduction, cardiomyocytes were transfected with mRNA encoding N-terminally FLAG-tagged Cas9. Four days after transfection, cells were fixed and immunostained. Scale bar: 50 μm. The proportion of tdTomato-positive cells among troponin I–positive cardiomyocytes was calculated using the image cytometry (n = 4, means ± SD).

In mammals, cardiomyocytes rapidly proliferate during fetal life, but exit the cell cycle soon after birth^27^. Although a recent report showed that AAV-mediated delivery of sgRNA can efficiently disrupt a target gene in adult cardiomyocyte expressing Cas9^28^, it remained uncertain whether HDR-mediated gene repair actually occurs in cardiomyocytes. To evaluate the efficiency of HDR in cardiomyocytes, we targeted myosin light chain 2 (Myl2), which constitutes the sarcomeric thick filament and is abundantly and specifically expressed in cardiomyocytes^29^. We designed and validated sgRNAs targeting the 3′ end of the *Myl2*-coding sequence (Fig. S2A) using single-strand annealing and mismatch-specific nuclease assays (Fig. S2B), and then selected the most effective sgRNA, sgRNA#2, which targeted the downstream region of the stop codon in exon 7 of *Myl2*. We next designed an HDR template including the tdTomato sequence flanked by the homology arms to generate Myl2-tdTomato fusion protein (Fig. 2A and Fig. S2C). A PAM mutation (CC to AA) was introduced in the 3′ homology arm to prevent Cas9-mediated cleavage. AAV serotype 6 (AAV6) has high tropism to cardiomyocytes^30, 31^; in our experiments in cultured neonatal cells, AAV6 encoding LacZ transduced approximately 60% of cardiomyocytes but <10% of non-cardiomyocytes (Fig. S2D). Purified AAV6 encoding sgRNA against *Myl2* and tdTomato HDR template (Fig. 2B) was transduced into cardiomyocytes isolated from Cas9 knock-in mice pre-seeded in 96-well plates. Four days after transduction, we observed cardiomyocytes positive for tdTomato fluorescent signals that clearly co-localized with sarcomeric structures detected by anti-troponin I antibody staining (Fig. 2C). Genomic PCR and Sanger sequencing confirmed precise genomic recombination at the *Myl2* locus (Fig. S2E and F).

The image cytometry enables sequential observation of a particular cell at specific coordinates in a 96-well plate. After 4 days of sequential observation of cultured cardiomyocytes transduced with AAV6, we found that weak tdTomato fluorescent signals appeared at day 2, and that fluorescence intensity gradually increased until day 4 (Fig. 2D). Although cardiomyocytes isolated from hearts during the neonatal stage have the capacity to enter mitotic phase^27^, sequential observation using the image cytometry detected HDR in a cardiomyocyte that did not migrate or divide during 4 days of observation (Fig. 2D and Fig. S2G). The proportion of tdTomato-positive cardiomyocytes gradually increased (Fig. S2H) and the sarcomeric structures in these cardiomyocytes (thin filament detected by Troponin I staining or Z lines detected by α-actinin staining) were not significantly affected by tagging of tdTomato fluorescent protein to C-terminus of Myl2 (Fig. S2I). In experiments using increasing viral titers, purified AAV6 dose-dependently increased the ratio of Myl2-tdTomato–positive cardiomyocytes and achieved ~20–25% HDR efficiency in cardiomyocytes constitutively expressing Cas9 (Fig. 2E and F). In western blots, Myl2-tdTomato fusion protein was detected at the expected molecular weight (Fig. S2J). The molar ratio of Myl2-tdTomato fusion protein estimated from western blot was 9.8%. To further detect HDR in cardiomyocytes isolated from wild type (WT) mice, we transfected mRNA encoding Cas9 12 h after the transduction of AAV6. Cas9 protein was transiently expressed in 12% of cardiomyocytes 12 h after transfection (Fig. 2G) and tdTomato-positive cardiomyocytes were detected at 3.2% among Troponin I-positive cardiomyocytes 4 days after transfection of mRNA (Fig. 2H). These data suggest that sequential observation of specific cardiomyocytes using the image cytometry is useful for evaluating the efficiency and time course of genome editing, and that HDR occurs in cardiomyocytes that do not enter mitotic phase.

To further detect HDR in adult cardiomyocytes, we isolated cardiomyocytes from adult hearts of 16–20 week old Cas9 knock-in mice using Langendorff perfusion method. Six h after isolation, AAV6 was transduced and the adult cardiomyocytes were incubated for 7 days. Six days after transduction, we could observe rod-shaped cardiomyocytes positive for red fluorescent signals (Fig. S2K). Genomic DNA was extracted 7 days after transduction. However, genomic PCR failed to detect tdTomato recombination, probably because of the low HDR efficiency and the low survival rate of adult cardiomyocytes under cultured conditions.

### S-phase entry is not necessarily required for HDR in cardiomyocytes

**Figure 3:**
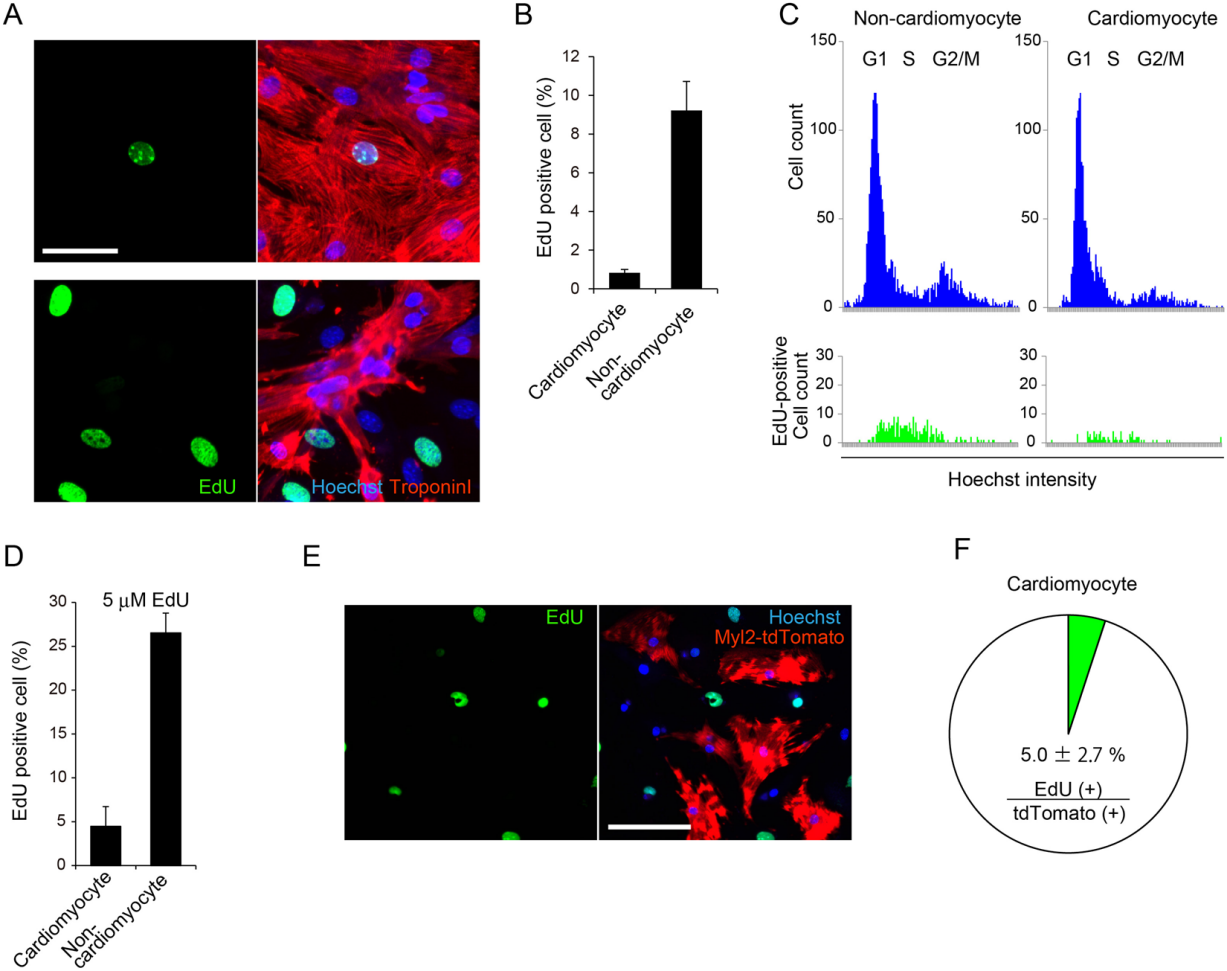
S-phase entry is not necessarily required for HDR in cardiomyocytes. (**A**) Two days after primary culture, cardiomyocytes and cardiac fibroblasts isolated from Cas9 knock-in mice were incubated in medium containing 5 μM EdU for 30 min, and then fixed. Cells were immunostained with anti–troponin I antibody. (**B**) Cardiomyocytes and fibroblasts seeded in 96-well plates were treated as in (**A**). The proportion of EdU-positive cardiomyocytes and fibroblasts were calculated using the image cytometry (n = 3, means ± SD). (**C**) Immunostaining images were obtained as in (B). DNA amount determined by Hoechst intensity in a total of 3,000 cardiomyocytes or fibroblasts were plotted as histograms. EdU-positive populations were highlighted in green in lower panels. (**D**) Cardiomyocytes and fibroblasts seeded in 96-well plates were treated with 5 μM EdU continuously for 4 days. Then cells were fixed and immunostained. The proportions of EdU-positive cardiomyocytes and fibroblasts were determined by image cytometry (n = 3, means ± SD). (**E**) Cardiomyocytes seeded in 96-well plates were transduced with AAV6 encoding HDR components 6 h after addition of EdU, followed by continuous labeling. After 4 days of culture, cells were fixed and immunostained. Representative images of cardiomyocytes positive for tdTomato without EdU staining are shown. Scale bar: 100 μm. (**F**) Cardiomyocytes were treated as in (**E**). The proportion of cardiomyocytes double-positive for both tdTomato and EdU among cardiomyocytes positive for tdTomato were calculated using the image cytometry (n = 3, means ± SD).

Cell-cycle synchronization experiments have suggested that S-phase entry is required for efficient HDR in cultured cells^22^. To determine whether HDR in cardiomyocytes is associated with cell-cycle phase, we evaluated the relationship between the occurrence of HDR and S-phase entry in cardiomyocytes. To this end, we analyzed immunostained cells on the image cytometry, enabling intensity-based evaluation of the cell cycle^32–34^. Specifically, we applied an algorithm to distinguish cardiomyocytes from non-cardiomyocytes as troponin I–positive cells segmented by the location of the nucleus^35^, and the nuclear DNA content in each cell was quantitatively analyzed using imaging software (Fig. S3A). Two days after primary culture, cardiac cells (including cardiomyocytes and non-cardiomyocytes) seeded in 96-well plates were labeled with EdU, a deoxyuridine analogue that is incorporated into genomic DNA during DNA synthesis^36^, for 30 min before fixation. As shown in Fig. 3A and B, ~8–10% of non-cardiomyocytes were positive for EdU, versus <1% of cardiomyocytes. The distribution of EdU-positive cells overlapped with the distribution of S-phase cells identified by intensity-based cell-cycle evaluation, suggesting that EdU labeling actually detected cardiomyocytes entering S-phase under our imaging-based experimental conditions (Fig. 3C). Next, to determine the cardiomyocytes that entered S-phase at least once during 4 days of culture, we continuously labeled the cells with EdU. After 4 days of labeling at 5 μM EdU, ~30% of non-cardiomyocytes and ~5% of cardiomyocytes were positive for EdU (Fig. 3D), suggesting that ~5% of cardiomyocytes entered S-phase at least once during 4 days of culture. To determine whether CRISPR/Cas9-mediated HDR occurred predominantly in cardiomyocytes that had passed through S-phase, we transduced cardiomyocytes with AAV6 encoding HDR components 6 h after addition of EdU, followed by continuous labeling for 4 days. As shown in Fig. 3E, after 4 days of labeling, most of the cardiomyocytes expressing Myl2-tdTomato were not positive for EdU. We observed that only ~5% of the cardiomyocytes were double-positive both for Myl2-tdTomato and EdU (Fig. S3B) among the cardiomyocytes positive for Myl2-tdTomato (Fig. 3F), suggesting that a large proportion of cardiomyocytes that underwent HDR had not entered S-phase. Although a previous report argued that cells need to pass through S phase in order to undergo efficient HDR^22^, our data obtained using continuous EdU labeling combined with sequential observation of individual cardiomyocytes suggest that S-phase entry is not necessary for HDR in these cells.

### HDR-mediated genome editing targeting a pathological deletion mutation in dilated cardiomyopathy (DCM) model mice

**Figure 4:**
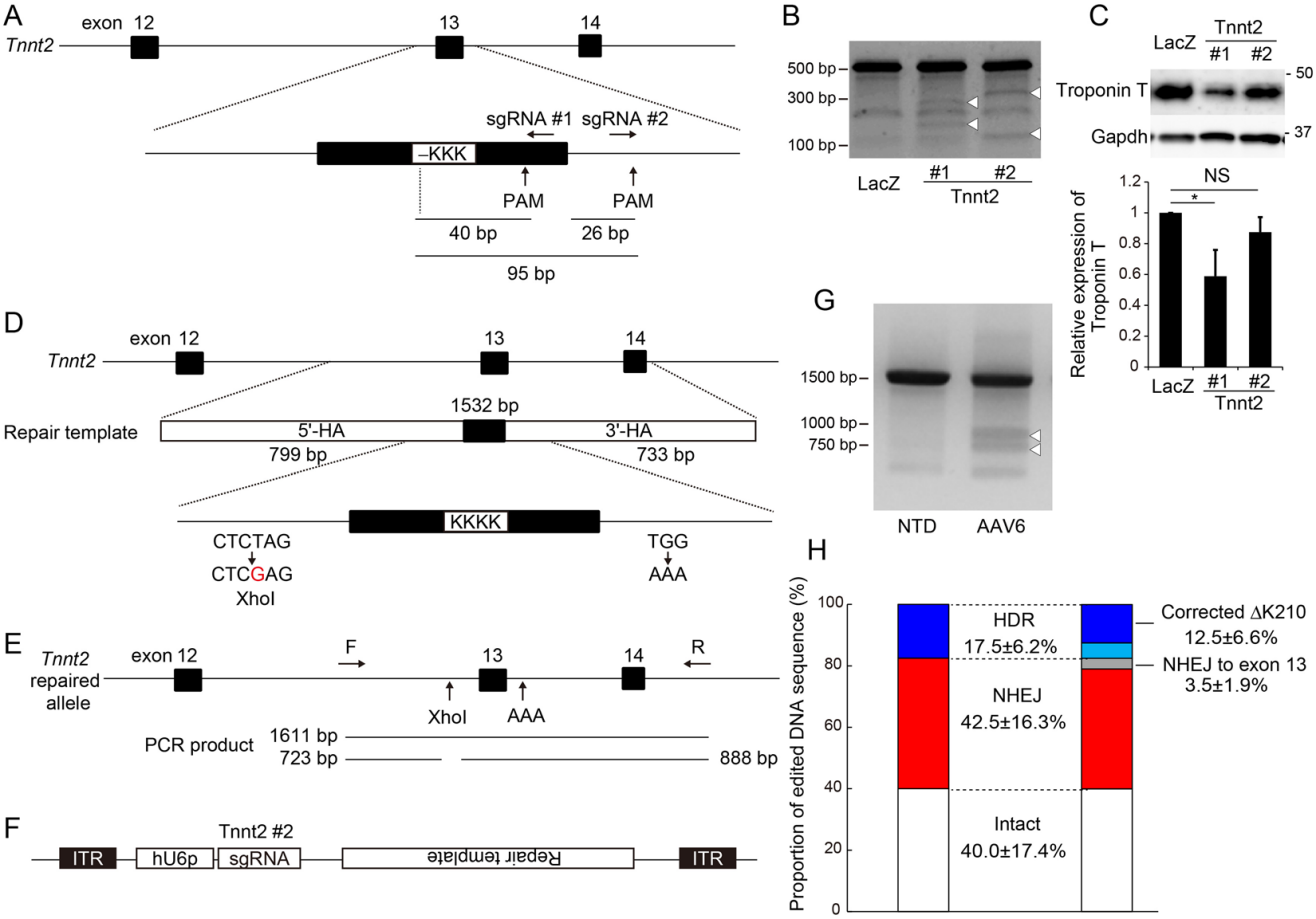
HDR-mediated genome editing targeting a pathological deletion mutation in dilated cardiomyopathy (DCM) model mice. (**A**) SgRNA-targeted site around exon 13 in the mouse *Tnnt2* gene. Distances from ΔK210 or 3′-terminal of exon 13 to each PAM sequence are shown. (**B**) Neonatal cardiomyocytes isolated from Cas9 knock-in mice were transfected with sgRNA against LacZ (control), or sgRNA #1 or #2 targeting *Tnnt2*. Forty-eight hours after transfection, genomic DNA was extracted, and cleavage activities were evaluated using the Cel-I assay. White arrowheads indicate cleaved PCR products. (**C**) Whole-cell lysates were extracted from the cells treated as in (**B**). Troponin T and Gapdh were detected by western blot. Expression level of Troponin T was normalized against the corresponding level of Gapdh (n = 3, means ± SD, *p < 0.05 vs LacZ). (**D**) Design of HDR repair template consisting of 799-bp 5′-terminal and 733-bp 3′-terminal homology arms (5′-HA and 3′-HA) corresponding to genomic sequence around exon 13 of *Tnnt2*. *Xho*I recognition site and PAM mutation (TGG to AAA) were introduced into both arms. (**E**) Detection of HDR by *Xho*I digestion of PCR products generated from primers inside (**F**) and outside (R) the homology arms. Expected lengths of PCR products are shown. (**F**) Design of AAV vector. hU6 promoter (hU6p)-driven sgRNA and HDR template were subcloned between inverse terminal repeat (ITR) sequences. (**G**) Neonatal cardiomyocytes isolated from Cas9 knock-in mice were transduced with AAV6 encoding editing components shown in (**F**) (1.88 × 10^5^ viral genomes/cell). Forty-eight hours after transfection, genomic DNA was extracted, and the targeted region was amplified by PCR. Purified PCR products were digested with *Xho*I. Arrowheads indicate the cleaved PCR products. NTD: non-transduced control. (**H**) PCR products shown in (**G**) were cloned and sequenced. Proportion of HDR, NHEJ (cleavage sites ranging or not ranging to exon 13), and intact sequence were evaluated (50 clones each, n = 4, means ± SD).

We next sought to apply HDR-mediated genome editing to genome repair targeting pathological mutations in cardiomyocytes of dilated cardiomyopathy (DCM) model mice. A deletion mutation of Lysine 210 (ΔΚ210) in the cardiac troponin T gene (*TNNT2*) was identified in familial DCM cases^37^, and mouse model harboring a knock-in of ΔΚ210 developed cardiac enlargement and heart failure, recapitulating the phenotypes of DCM patients^38^. In ΔK210 knock-in mice, the three bases (AAG) encoding K210 in exon 13 were deleted by homologous recombination. First, to cleave the neighboring region of ΔK210, we selected two sgRNAs, one targeting the exonic region (#1, PAM sequence located 40 bp from K210), and the other targeting the intronic region (#2, PAM sequence locates 95 bp from K210 and 26 bp from exon 13) to avoid cleavage of the exon (Fig. 4A). To evaluate the efficiency of genomic cleavage and the influence on protein expression of cardiac troponin T, each sgRNA was transfected into cultured neonatal cardiomyocytes isolated from Cas9 knock-in mice. As shown in Fig. 4B, both sgRNAs efficiently cleaved their genomic targets. Importantly, sgRNA #1 targeting exon 13 of *Tnnt2* significantly decreased protein expression levels, whereas sgRNA #2 did not (Fig. 4C). Because Cas9-mediated genomic deletions range in size from several base pairs to occasionally more than one hundred base pairs^39^, the 26 bp distance between exon 13 and the PAM sequence targeted by sgRNA #2 seems insufficient to completely avoid the influence of exon cleavage. Furthermore, the retention rate of an integrated sequence after HDR is negatively correlated with the distance between the sequence and the cleavage site^40, 41^. Nonetheless, our data suggest that specific targeting at an intronic region can minimize the deleterious effect of genomic cleavage on protein expression, prompting us to use sgRNA #2 for further experiment. Next, we constructed a 1532-bp repair template including K210, the PAM site mutation (TGG to AAA), and single-nucleotide mutation (T to G) to generate an *Xho*I recognition site at the intron upstream of exon 13 (Fig. 4D). PCR amplification using a primer pair targeting inside and outside the homology arm yielded a 1611-bp PCR product, and successful recombination was confirmed based on detection of the 723 and 888 bp fragments generated by *Xho*I digestion (Fig. 4E). These methods were initially validated using C2C12 cells co-transfected with pX459 and repair template plasmids (Fig. S4A). We crossed ΔK210 knock-in mice with Cas9 knock-in mice to generate double–knock-in mice homozygous for the ΔK210 allele and constitutively expressing Cas9 (Fig. S4B). We then generated AAV6 encoding sgRNA against *Tnnt2* (#2) and repair template (Fig. 4F), and transduced neonatal cardiomyocytes isolated from double knock-in mice. Forty-eight hours after transduction, we extracted genomic DNA, performed PCR, and digested the PCR products with *Xho*I. As shown in Fig. 4G, cleaved PCR products were observed in genomic DNA samples isolated from cardiomyocytes treated with AAV6. Transduction with AAV6 encoding sgRNA and repair template did not significantly affect the protein expression levels of Tnnt2 in cardiomyocytes (Fig. S4C). Sequence analysis of 50 clones per sample (each sample was isolated from an individual neonatal heart; biological replicates = 4) revealed that AAV6-mediated genome repair achieved HDR at a rate of 17.5% (Fig. 4H and S4D). Correct repair of ΔK210 to yield the WT sequence was observed at a rate of 12.5%, whereas in the other clones, genomic repair via HDR was limited to the region around the PAM sequence and did not reach the ΔK210 sequence (Fig. 4H and S4D). On the other hand, genome repair resulted in 42.5% of NHEJ. Importantly, Cas9-mediated cleavage extending to exon 13 was observed at a frequency of only 3.5%. In 40% of cases the genome remained unchanged, probably because of the limited transduction efficiency of AAV6 in cardiomyocytes under our experimental conditions (Fig. S2D). These data suggest that AAV6-mediated genome editing specifically avoiding exonic regions can be useful for repairing mutated alleles in cardiomyocytes from DCM model mice.

## Discussion

In the present study, we demonstrated that HDR-mediated genome editing using CRISPR/Cas9 is effective in non-dividing cardiomyocytes by sequential observation of individual cardiomyocytes using an image cytometry. Cell sorting analysis and detection of fluorescent signals from an integrated fluorescent protein have been used previously for quantitative assessment of HDR^42–44^. Although these methods are useful, selectively sorting cardiomyocytes from inhomogeneous primary cultured cardiac cells is technically difficult, necessitating another analytical method. The image cytometry enabled the detection of HDR specifically occurring in cardiomyocytes, distinguished by Troponin I staining and detection of the precise recombination of the *Myl2* gene resulting in proper localization of the protein at sarcomeric structures. Furthermore, the behavioral characteristics of cardiomyocytes, most of which do not migrate after primary culture, enabled sequential observation of time-course changes of gene-edited cells. In this manner, we discovered that HDR in cardiomyocytes was independent of DNA synthesis. Although HDR is restricted to late S and G2 phases, when DNA replication has been completed and sister chromatids are available to serve as repair templates^21^, our results suggest that exogenously provided precise template DNA can promote HDR-mediated genome repair in non-dividing cardiomyocytes. Importantly, genomic recombination via HDR was observed in cardiomyocytes at neonatal stage but not in adult cardiomyocytes, suggesting that further improvements are required to translate these methods to adult cells.

For the purpose of replacing genomic sequence in non-dividing cells, NHEJ-mediated methods have been developed in which integrated sequences in template DNA are excised by Cas9 and ligated at the DSB site under the guidance of microhomology^45–47^. More recently, homology-independent targeted integration was reported as a novel method for efficiently replacing genomic sequence in non-dividing neurons^48^. These editing methods are principally based on NHEJ, which is error-prone compared to HDR^12–17^. Our HDR-based editing method achieved approximately 12.5% of precise correction of mutated sequence in cardiomyocytes treated with genome editing components, and thus yielded higher efficiency than an NHEJ-based method in primary neurons^48^. Although the HDR-mediated method provided accurate and efficient results, the remaining 40% deleterious NHEJ-mediated mutations must be repressed via pharmacological or biological interventions in order for this approach to be used therapeutically^42, 49^. Furthermore, because our AAV-mediated correction method used cardiomyocytes constitutively expressing Cas9, therapeutic applications would require an efficient method for delivering Cas9 into cardiomyocytes. Efficient *in vivo* delivery method have been developed using AAV encoding Cas9 driven by a minimized promoter^10^ or the smaller Cas9 orthologue from *Staphylococcus aureus* (SaCas9)^11^. The combination of Cas9 mRNA and AAV encoding sgRNA and the repair template is both useful and safe, enabling complete removal of the nuclease from the edited cells^8^. Taken together, our findings suggest that HDR-mediated genome replacement represents a promising therapeutic tool for targeting intractable cardiomyopathy resulting from pathological mutation.

## Methods

### Cell Culture

HEK293T cells and C2C12 cells were maintained in high glucose Dulbecco′s Modified Eagle Medium (DMEM, Gibco) containing 10% fetal bovine serum (FBS, Gibco) and penicillin, streptomycin and glutamine (PSG, Gibco). Neonatal mouse cardiomyocytes were prepared as previously described with modification^50^. In brief, Harvested hearts were incubated in 0.025% trypsin/EDTA (Sigma) at 4 °C overnight and then digested with collagenase type II (Worthington). The cardiomyocyte fraction was collected after differential plating for 70 min at 37 °C, seeded and incubated with high glucose DMEM with 10% fetal bovine serum and PSG. The adhering cardiac fibroblast were separately incubated and used for experimental analysis after more than one passage as previously described^26^. All procedures were performed in conformity with the *Guide for the Care and Use of Laboratory Animals published by the US National Institutes of Health* (NIH Publication, 8th Edition, 2011) and were approved by the Osaka University Committee for Laboratory Animal Use.

### HDR constructs

DNA sequences for 5′-terminal homology arm (5′-HA) and 3′-terminal homology arm (3′-HA) were amplified from genomic DNA using the primer pairs including recognition sites of restriction enzymes. DNA sequences for tdTomato fluorescent proteins was amplified from pCSCMV:tdTomato vector (Addgene #30530). Each DNA fragment was digested by restriction enzymes and cloned into pUC19 vector (TaKaRa) to generate HDR template. hU6 promoter and downstream single guide RNA (sgRNA) sequence was amplified from pX459 vector (Addgene #48139). hU6 promoter sequence, sgRNA sequence and HDR template sequence were cloned into between inverse terminal repeat (ITR) sequences in pAAV vector (TaKaRa). PAM sequence mutation was introduced into each HDR template to avoid Cas9-mediated cleavage. In HDR template for *Tnnt2* gene, XhoI recognition sequence was introduced into intronic sequence just upstream of exon containing pathological mutation (ΔK210). sgRNAs and primer sequences are listed in Supplementary Table.

### HDR detection using image cytometry

Cardiac fibroblasts were seeded in Greiner CELLSTAR 96 well plates (5,000 cells/well) one day before transduction with AAV encoding sgRNA and repair template. Cardiomyocytes (2 × 10^4^ cells/well) were seeded in Greiner CELLSTAR 96 well plates which were previously coated with collagen (Celmatrix Type I-C (KURABO)). Twenty-four hours after primary culture, AAV encoding sgRNA and repair template were transduced. Twelve hours after transduction, medium were exchanged and cells were incubated for the indicated time. For sequential observation, fluorescent images from living cardiomyocytes cultured in 96 well plates were obtained by IN Cell Analyzer 6000 (GE). After sequential observation, cells were fixed, immunostained and fluorescent images were obtained with the same imaging acquisition protocol. A total of 64 nonoverlap images (16 images per well) were obtained from each sample in one experiment using a 20 × /0.45NA Nikon lens. Bright field and fluorescent images at the specific coordinate in 96 well plates were shown. The proportion of HDR was calculated as the ratio of tdTomato-positive cells per total cardiac fibroblasts or total cardiomyocytes using IN Cell Developer Toolbox (GE). Detailed Analytical procedures for recognition, segmentation of the cells are shown in Fig. S3A.

### EdU labeling and intensity-based cell-cycle evaluation

Cardiomyocytes (2 × 10^4^ cells/well) were seeded in Greiner CELLSTAR 96 well plates which were previously coated with collagen (Celmatrix Type I-C (KURABO)). EdU at the indicated concentration was added to the culture medium from a 100 mM stock in DMSO. After EdU labeling for the indicated time, cells were washed with PBS and fixed. The labeled EdU of the cells were visualized by Click-iT imaging kits (Life Technology) according to the manufacturer’s instructions. The samples were incubated with the working solution of Click-iT reaction cocktail, containing the Alexa Fluor 488 azide and CuSO_4_, for 30 min at room temperature. After removal of the reaction cocktail, cells were washed with Click-iT reaction rinse buffer and PBS, then transferred to immunostaining procedures. After incubation with blocking buffer (1% BSA in PBS), the cells were immunostained with anti-cardiac Troponin I polyclonal antibody for 1 h at room temperature. After washing with PBS, the cells were incubated with a goat anti–rabbit IgG secondary antibody, conjugated with Alexa Fluor 568 (Invitrogen) diluted by blocking buffer containing Hoechst. The immunofluorescent images were obtained using IN Cell Analyzer 6000. A total of 64 nonoverlap images (16 images per well) were obtained from each sample in one experiment using a 20 × /0.45NA Nikon lens. The obtained images were analyzed using IN Cell Developer toolbox (version1.9, GE). Individual DNA contents of each cardiomyocyte or non-cardiomyocyte were calculated as fluorescent signals from Hoechst staining (density x area) and plotted as histograms. EdU-positive cells were plotted as the cells during S-phase. The average data were obtained from at least three biological replicates.

### Generation and purification of AAV

HEK293T cells were transfected with the vector of interest, pHelper vector and pRC2 or pRC6 Vector (AAVpro Helper Free System, TaKaRa) using calcium phosphate transfection (CalPhos Mammalian Transfection Kit,TaKaRa). Seventy-two hours after transfection, HEK293T cells were detached by addition of 1/80 volume of 0.5M EDTA (pH 8.0), then pelleted via low-speed centrifugation (2000 × g for 10 min). Cell pellet was lysed with AAV Extraction Solution A and centrifuged (9000 × g for 10 min). AAV Extraction Solution B was added to the collected to supernatant and stored at −80 °C. Collected AAV generated from HEK293T cells (8–9 × 10^7^ cells) was purified using AAVpro Purification Kit (TaKaRa), and viral titer was calculated using AAV Titration Kit (TaKaRa).

## Electronic supplementary material

Supplementary Information

Supplemental Table

## References

[1] Allen LA (2012). Decision making in advanced heart failure: a scientific statement from the American Heart Association. Circulation.

[2] Yancy CW (2013). 2013 ACCF/AHA guideline for the management of heart failure: a report of the American College of Cardiology Foundation/American Heart Association Task Force on practice guidelines. Circulation.

[3] Herman DS (2012). Truncations of titin causing dilated cardiomyopathy. N Engl J Med.

[4] Haas J (2015). Atlas of the clinical genetics of human dilated cardiomyopathy. Eur Heart J.

[5] Ware JS (2016). Shared Genetic Predisposition in Peripartum and Dilated Cardiomyopathies. N Engl J Med.

[6] Cox DB, Platt RJ, Zhang F (2015). Therapeutic genome editing: prospects and challenges. Nat Med.

[7] Lin SR (2014). The CRISPR/Cas9 System Facilitates Clearance of the Intrahepatic HBV Templates *In Vivo*. Mol Ther Nucleic Acids.

[8] Yin H (2016). Therapeutic genome editing by combined viral and non-viral delivery of CRISPR system components *in vivo*. Nat Biotechnol.

[9] Long C (2014). Prevention of muscular dystrophy in mice by CRISPR/Cas9-mediated editing of germline DNA. Science.

[10] Long C (2016). Postnatal genome editing partially restores dystrophin expression in a mouse model of muscular dystrophy. Science.

[11] Tabebordbar M (2016). *In vivo* gene editing in dystrophic mouse muscle and muscle stem cells. Science.

[12] Mikuni T, Nishiyama J, Sun Y, Kamasawa N, Yasuda R (2016). High-Throughput, High-Resolution Mapping of Protein Localization in Mammalian Brain by *In Vivo* Genome Editing. Cell.

[13] Doudna JA, Charpentier E (2014). Genome editing. The new frontier of genome engineering with CRISPR-Cas9. Science.

[14] Hsu PD, Lander ES, Zhang F (2014). Development and applications of CRISPR-Cas9 for genome engineering. Cell.

[15] Sander JD, Joung JK (2014). CRISPR-Cas systems for editing, regulating and targeting genomes. Nat Biotechnol.

[16] Chapman JR, Taylor MR, Boulton SJ (2012). Playing the end game: DNA double-strand break repair pathway choice. Mol Cell.

[17] Iyama T, Wilson DM (2013). DNA repair mechanisms in dividing and non-dividing cells. DNA Repair (Amst).

[18] Rothkamm K, Kruger I, Thompson LH, Lobrich M (2003). Pathways of DNA double-strand break repair during the mammalian cell cycle. Mol Cell Biol.

[19] Sharma S (2007). Age-related nonhomologous end joining activity in rat neurons. Brain Res Bull.

[20] Ciccia A, Elledge SJ (2010). The DNA damage response: making it safe to play with knives. Mol Cell.

[21] Heyer WD, Ehmsen KT, Liu J (2010). Regulation of homologous recombination in eukaryotes. Annu Rev Genet.

[22] Lin S, Staahl BT, Alla RK, Doudna JA (2014). Enhanced homology-directed human genome engineering by controlled timing of CRISPR/Cas9 delivery. Elife.

[23] Platt RJ (2014). CRISPR-Cas9 knockin mice for genome editing and cancer modeling. Cell.

[24] Hsu PD (2013). DNA targeting specificity of RNA-guided Cas9 nucleases. Nat Biotechnol.

[25] Mashiko D (2013). Generation of mutant mice by pronuclear injection of circular plasmid expressing Cas9 and single guided RNA. Sci Rep.

[26] Takeda N (2010). Cardiac fibroblasts are essential for the adaptive response of the murine heart to pressure overload. J Clin Invest.

[27] Ahuja P, Sdek P, MacLellan WR (2007). Cardiac myocyte cell cycle control in development, disease, and regeneration. Physiol Rev.

[28] Carroll KJ (2016). A mouse model for adult cardiac-specific gene deletion with CRISPR/Cas9. Proc Natl Acad Sci USA.

[29] Tsukamoto O, Kitakaze M (2013). Biochemical and physiological regulation of cardiac myocyte contraction by cardiac-specific myosin light chain kinase. Circ J.

[30] Zincarelli C, Soltys S, Rengo G, Rabinowitz JE (2008). Analysis of AAV serotypes 1-9 mediated gene expression and tropism in mice after systemic injection. Mol Ther.

[31] Neuber C (2014). Paradoxical effects on force generation after efficient beta1-adrenoceptor knockdown in reconstituted heart tissue. J Pharmacol Exp Ther.

[32] Poon SS (2008). Intensity calibration and automated cell cycle gating for high-throughput image-based siRNA screens of mammalian cells. Cytometry A.

[33] Futakuchi A (2016). The effects of ripasudil (K-115), a Rho kinase inhibitor, on activation of human conjunctival fibroblasts. Exp Eye Res.

[34] Toledo LI (2013). ATR prohibits replication catastrophe by preventing global exhaustion of RPA. Cell.

[35] Masumura Y (2016). Btg2 is a Negative Regulator of Cardiomyocyte Hypertrophy through a Decrease in Cytosolic RNA. Sci Rep.

[36] Salic A, Mitchison TJ (2008). A chemical method for fast and sensitive detection of DNA synthesis *in vivo*. Proc Natl Acad Sci USA.

[37] Kamisago M (2000). Mutations in sarcomere protein genes as a cause of dilated cardiomyopathy. N Engl J Med.

[38] Du CK (2007). Knock-in mouse model of dilated cardiomyopathy caused by troponin mutation. Circ Res.

[39] Tsai SQ (2015). GUIDE-seq enables genome-wide profiling of off-target cleavage by CRISPR-Cas nucleases. Nat Biotechnol.

[40] Kan Y, Ruis B, Lin S, Hendrickson EA (2014). The mechanism of gene targeting in human somatic cells. PLoS Genet.

[41] Paquet D (2016). Efficient introduction of specific homozygous and heterozygous mutations using CRISPR/Cas9. Nature.

[42] Chu VT (2015). Increasing the efficiency of homology-directed repair for CRISPR-Cas9-induced precise gene editing in mammalian cells. Nat Biotechnol.

[43] Ratz M, Testa I, Hell SW, Jakobs S (2015). CRISPR/Cas9-mediated endogenous protein tagging for RESOLFT super-resolution microscopy of living human cells. Sci Rep.

[44] Kimura Y (2015). CRISPR/Cas9-mediated reporter knock-in in mouse haploid embryonic stem cells. Sci Rep.

[45] Sakuma T, Nakade S, Sakane Y, Suzuki KT, Yamamoto T (2016). MMEJ-assisted gene knock-in using TALENs and CRISPR-Cas9 with the PITCh systems. Nat Protoc.

[46] Nakade S (2014). Microhomology-mediated end-joining-dependent integration of donor DNA in cells and animals using TALENs and CRISPR/Cas9. Nat Commun.

[47] Hisano Y (2015). Precise in-frame integration of exogenous DNA mediated by CRISPR/Cas9 system in zebrafish. Sci Rep.

[48] Suzuki K (2016). *In vivo* genome editing via CRISPR/Cas9 mediated homology-independent targeted integration. Nature.

[49] Maruyama T (2015). Increasing the efficiency of precise genome editing with CRISPR-Cas9 by inhibition of nonhomologous end joining. Nat Biotechnol.

[50] Shintani Y (2014). Toll-like receptor 9 protects non-immune cells from stress by modulating mitochondrial ATP synthesis through the inhibition of SERCA2. EMBO Rep.

