# Supplementary material for: Targeted Genome Replacement via Homology-directed Repair in Non-dividing Cardiomyocytes
Source: Sci Rep. 2017 Aug 24;7:9363. doi: 10.1038/s41598-017-09716-x (PMC5571012; doi:10.1038/s41598-017-09716-x)
Supplement: Supplementary file 1 — Supplementary Information [file 41598_2017_9716_MOESM1_ESM.pdf]

Supplemental Information

Title

Targeted Genome Replacement via Homology-directed Repair in Non-dividing Cardiomyocytes

Authors/Affiliations

Takamaru Ishizu[1], Shuichiro Higo[1], Yuki Masumura[1], Yasuaki Kohama[1], Mikio Shiba[1], Tomoaki Higo[1], Masato Shibamoto[1], Akito Nakagawa[1], Sachio Morimoto[2], Seiji Takashima[3], Shungo Hikoso[1,4] and Yasushi Sakata[1]

[1] Department of Cardiovascular Medicine, Osaka University Graduate School of Medicine, Suita, Osaka 565-0871, Japan

[2] Department of Health and Medical Care, International University of Health and Welfare, Okawa, Fukuoka 831-8501, Japan

[3] Department of Medical Biochemistry, Osaka University Graduate School of Medicine, Suita, Osaka 565-0871, Japan

[4] Department of Medical Therapeutics for Heart Failure, Osaka University Graduate School of Medicine, Suita, Osaka 565-0871, Japan

Supplemental Methods

Reagents and antibodies

Antibodies used in this study: Cardiac Troponin I (Abcam Cat# ab47003 RRID:AB_869982), Gapdh (Millipore Cat# MAB374 RRID:AB_2107445), α-SMA (Sigma-Aldrich Cat# A2547 RRID:AB_476701), Vimentin (Abcam Cat# ab24525 RRID:AB_778824), RFP-HRP (clone 1G9, MBL), FLAG M2 antibody (Sigma-Aldrich Cat# F1804, RRID:AB_262044), α-actinin (Abcam Cat# ab9465, RRID:AB_307264), β-actin (Actb) (Cell Signaling Technology Cat# 4967, RRID:AB_330288), Myl2 (Proteintech Group Cat# 10906-1-AP RRID:AB_2147453). Alexa 488-, 568- and 647-conjugated secondary antibodies (Invitrogen).

Single strand annealing assays

Targeted genomic sequences were cloned into pCAG-EGxxFP vector [1] encoding tandem truncated EGFP gene with overlapped sequence separated by the cloning site. pCAG-EGxxFP vector with or without pX459 vector [2] encoding SpCas9 and the indicated sgRNAs were transfected into 293T cells pre-seeded in Greiner CELLSTAR 96 well plate (1×10^4 cells/well). Forty-eight hours after transfection, the fluorescent images of EGFP were obtained by image cytometry, and quantitatively analyzed using IN Cell Developer Toolbox (GE). A total of 36 nonoverlap images (9 images per well) were obtained from each sample in one experiment using a 10×/0.45NA Nikon lens.

Cel-I assay

C2C12 cells were seeded in 12 well plate (2×10^4 cells/well) one day before transfection. pX459 vector was transfected into C2C12 cells using Lipofectamine 3000 (Life Technologies). Two days after transfection, medium was exchanged to the medium containing 3 μg/mL puromycin to select the cells expressing Cas9. After puromycin selection, genomic DNA were extracted using QIAamp DNA Mini Kit (QIAGEN). Neonatal mouse cardiomyocytes isolated from Cas9 knock-in mice were seeded in 24 well plate (5×10^5 cells/well) one day before transfection. Five hundred ng of *Tnnt2* sgRNA#1 or #2 was transfected into neonatal mouse cardiomyocytes using Lipofectamine

MessengerMAX (Life Technologies). Two days after transfection, genomic DNA were extracted. Target regions were amplified by PCR (KOD Fx Neo, TOYOBO) as follows: 94 °C for 2 min, followed by 30 cycles of 98 °C for 10 s, annealing temperature (depending on primer sequences) for 30 s and 68 °C for 30 s. Primer sequences are listed in Supplementary Table. After purification of PCR products using QIAquick PCR purification kit (QIAGEN), PCR fragments both from untreated and treated allele were hybridized to form hetero DNA duplex. Then, hybridized PCR hetero duplexes were enzymatically digested by mismatch-specific endonuclease, Cel-I 42 °C for 60 min (SURVEYOR Mutation Detection Kit).

Genotyping

Genomic DNA were extracted from mouse tail using a ready-made extraction solution (Express Extract, KAPA Biosystems). PCR reactions were performed using 5 μl ReadyMix (HiFi HotStart ReadyMix, KAPA Biosystems), 0.5 μl of 10 μM forward and reverse primers, 0.4 μl of DNA extract and PCR-grade water to a total amount of 10 μl. For Cas9 KI mice, 10 μM forward and two reverse primers were mixed. PCR conditions are as follows: 95 °C for 3 min, followed by 33 cycles of 95 °C for 10 s, 60 °C for 10 s and 72 °C for 10s. Amplified DNA was separated by electrophoresis in a 2 % TBE agarose gel.

Western Blotting

For western blotting, cells were washed with cold PBS and directly lysed with SDS buffer (50mM Tris-HCl (pH7.4), 5mM EDTA). The protein concentration was determined when required by BCA Protein Assay Kit (Thermo). Lysate samples were mixed with 4 × Laemmli sample buffer (BioRad) with mercaptoethanol (2.5%). Proteins were separated by SDS-PAGE and transferred to PVDF membrane. Antibodies were diluted by 3% nonfat milk. After blocking with 3% nonfat milk for 1 h, the transferred membrane was incubated with primary antibody at 4°C overnight and with secondary antibody at room temperature for 30 min. The membrane signals were detected by chemiluminescence using ECL or ECL prime reagent (GE). Gapdh was used as control for equal loading and transfer. The protein expression level was quantified using ImageQuant TL (GE). Theoretical

molecular weight of the protein was calculated using website tool (ExPASy: http://web.expasy.org/compute_pi/). The number of moles of each protein band were estimated by normalizing protein expression levels by molecular weight and relative molar ratio of tdTomato-tagged protein was calculated.

Generation of sgRNA, Cas9 mRNA and transfection

sgRNAs were generated by MEGAshortscript T7 Transcription Kit (Life Technologies) using custom-made double strand oligo DNA nucleotide (GeneArt CRISPR T7 strings DNA, Life Technologies) as a template. sgRNA targeting LacZ was used as a control [3]. The Cas9 mRNA was transcribed using FLAG-tagged Cas9 expression vector and the mMESSAGE mMACHINE T7 ULTRA kit (Life Technologies). After in vitro transcription, DNase I treatment and poly (A) tailing reaction were performed. The generated RNAs were then purified using MEGA clear kit (Life Technologies). Purified sgRNAs or mRNA was transfected into neonatal cardiomyocytes using Lipofectamine messenger MAX (Life Technologies).

Langendorff-perfusion of adult mouse heart

The adult hearts were quickly excised and mounted onto a Langendorff perfusion apparatus as previously reported [4]. The hearts were perfused with calcium-free buffer (0-Ca^{2+} buffer: 126 mM NaCl, 8.8 mM KCl, 2.0 mM $MgCl_2$, 24 mM HEPES, 5.0 mM sodium pyruvate, 11 mM D-glucose, 1 × Glutamax, 30 mM 2,3-Butanedione monoxime, 4.4 mM creatine monohydrate, 8.0 mM Taurine, pH 7.4) for 5 min at 2 ml/min, then with collagenase solution (1 mg/ml type 2 collagenase, 0.2 mg/ml protease type 14, 50 μM $CaCl_2$ in 0-Ca^{2+} buffer) for 10 min at 3 ml/min. The whole system was maintained at 39 °C. Following perfusion, hearts were placed in a 60-mm dish containing 50 μM Ca^{2+} buffer (50 μM $CaCl_2$ in 0-Ca^{2+} buffer) and minced with micro-scissors into small pieces. Non-cardiomyocytes were quickly removed according to their size (smaller than cardiomyocytes) using cell strainer. Isolated cardiomyocytes were incubated for 7 days after AAV6 transduction.

Statistical Analysis

Data are expressed as means ± S.D. of at least three independent experiments, unless otherwise indicated. The two-tailed Student's *t*-test or one-way ANOVA with repeated measures followed by Post-hoc Tukey test were used to analyze differences between two groups. *p*-value < 0.05 was considered statistically significant.

Supplemental Reference

1 Mashiko, D. *et al.* Generation of mutant mice by pronuclear injection of circular plasmid expressing Cas9 and single guided RNA. *Sci Rep* **3**, 3355, doi:10.1038/srep03355 (2013).

2 Ran, F. A. *et al.* Genome engineering using the CRISPR-Cas9 system. *Nat Protoc* **8**, 2281-2308, doi:10.1038/nprot.2013.143 (2013).

3 Platt, R. J. *et al.* CRISPR-Cas9 knockin mice for genome editing and cancer modeling. *Cell* **159**, 440-455, doi:10.1016/j.cell.2014.09.014 (2014).

4 Higo, T. *et al.* DNA single-strand break-induced DNA damage response causes heart failure. *Nat Commun* **8**, 15104, doi:10.1038/ncomms15104 (2017).

5 Du, C. K. *et al.* Knock-in mouse model of dilated cardiomyopathy caused by troponin mutation. *Circ Res* **101**, 185-194, doi:10.1161/CIRCRESAHA.106.146670 (2007).

Supplemental Figure Legends

Figure S1

A) Cleavage activity of each sgRNA targeting exon 6 of mouse *Actb* was evaluated by single-strand annealing assay (see Methods) (n=3, means ± SD).

B) C2C12 cells were transfected with pX459 vector encoding sgRNA against exon 6 of mouse *Actb*. Two days after transfection, cells were exposed to 3 μg/mL puromycin for two days. Genomic DNA was extracted, and cleavage activities were evaluated by Cel-I assay. White arrowhead indicates the cleaved PCR products.

C) The nucleotide sequence and its translated amino acid sequence at the 3'-terminal region of *Actb*. The expected sequences after HDR are shown below.

D) Isolated cardiac fibroblasts were immunostained with anti–α-smooth muscle actin (α-SMA) or anti-vimentin antibodies. Nuclei were stained with Hoechst. Scale bar: 100 μm.

E) PCR products shown in Fig. 1F were cloned and sequenced. Electropherograms of recombination sites are shown.

F) Cardiac fibroblasts isolated from Cas9 knock-in mice were transduced with AAV2 encoding sgRNA and HDR template (3.02 x 10^4 viral genomes/cell). Forty-eight hours after transduction, whole-cell lysates were prepared and analyzed by western blot using anti-Actb and anti-RFP antibodies. White and black arrowheads indicate the Actb-tdTomato fusion protein and endogenous Actb, respectively. The estimated molar ratio of Actb-tdTomato fusion protein and endogenous Actb is shown (n=3, means ± SD). GAPDH was used as a loading control.

G) Cardiac fibroblasts isolated from Cas9 knock-in mice were seeded in 96-well plates and transduced with AAV2 encoding sgRNA and HDR template (9.1×10^3 viral genomes/cell). Two, 4 and 6 days after transduction, cells were fixed and stained with anti-Vimentin and anti–α-SMA antibody. The proportion of tdTomato-positive fibroblasts (among Vimentin-positive cells) was calculated using the image cytometry (n=3, means ± SD, *: $p<0.05$ vs day 2)

H) Cardiac fibroblasts isolated from Cas9 knock-in mice were treated as in (G). Six days after transduction, cells were passaged and incubated until day 12, then fixed and stained as in (G). Representative fluorescent images

at day 6 and day 12 are shown. Scale bar: 50 μm.

Figure S2

A) Four candidate sgRNAs were designed to cleave near the stop codon in exon 7 of the mouse *Myl2* gene.

B) Cleaving activity of each sgRNA targeting exon 7 of *Myl2* was evaluated by single-strand annealing assay (left, n=3, means ± SD). C2C12 cells were transfected with pX459 vector encoding sgRNA against *Myl2* (#2). Two days after transfection, cells were exposed to 3 μg/mL puromycin for 2 days. Genomic DNA was extracted, and cleavage activities were evaluated by Cel-I assay (right).

C) The nucleotide sequence and its translated amino acid sequence at the 3'-terminal region of *Myl2*. The expected sequences after HDR are shown below.

D) Cardiac cells isolated from neonatal hearts were transduced with AAV6 encoding LacZ at increasing viral titer (1.93×10^5, 4.83×10^5, 9.66×10^5 viral genomes/cell). Two days after transduction, cells were fixed and immunostained with anti-LacZ and anti–Troponin I antibodies. The proportions of LacZ-positive cells among Troponin I–positive cardiomyocytes and Troponin I–negative non-cardiomyocytes were evaluated using the image cytometry (n=3, means ± SD).

E) Cardiomyocytes isolated from Cas9 knock-in mice were seeded in 96-well plates and transduced with AAV serotype 6 (AAV6) encoding sgRNA and HDR template. Four days after transduction, genomic DNA was extracted, and genomic PCR was performed using the indicated primers (shown in Fig. 2A). NTD: non-transduced control.

F) PCR products shown in (E) were cloned and sequenced. Electropherograms at recombination sites are shown.

G) Cardiomyocytes isolated from Cas9 knock-in mice were seeded in 96-well plates and transduced with AAV serotype 6 (AAV6) encoding sgRNA and HDR template. After transduction of AAV, bright-field and fluorescence images were sequentially obtained using the image cytometry targeting specified fields determined by coordinate axes. On day 4, cells were fixed and immunostained. Representative images are shown. The top panels are the time course of the edited cardiomyocytes shown in Fig. 2C. Arrowheads indicate the tdTomato-

positive cardiomyocytes. Scale bar: 100 μm.

H) Cardiomyocytes isolated from Cas9 knock-in mice were seeded in 96-well plates and transduced with AAV6 encoding sgRNA and HDR template (2.17×10^4 viral genomes/cell). Two, 4 and 6 days after transduction, cells were fixed and stained with anti-Troponin I antibody. The proportion of tdTomato-positive cells among Troponin I–positive cardiomyocytes was calculated using the image cytometry. (n=3, means ± SD, *: $p<0.05$ vs day 2).

I) Six days after transduction, cardiomyocytes treated as in (H) were fixed and immunostained with anti-Troponin I and anti-α-actinin antibody. Sarcomeric structure in tdTomato-positive cardiomyocytes in white square are enlarged in lower panels. Scale bar: 50 μm.

J) Cardiomyocytes isolated from Cas9 knock-in mice were transduced with AAV6 encoding sgRNA and HDR template (2.17×10^4 viral genomes/cell). Three days after transduction, whole-cell lysates were prepared and analyzed by western blot using anti-Myl2 and anti-RFP antibodies. White and black arrowheads indicate the Myl2-tdTomato fusion protein and endogenous Myl2, respectively. The estimated molar ratio of Myl2-tdTomato fusion protein and endogenous Myl2 is shown (n=3, means ± SD). GAPDH was used as a loading control.

K) Cardiomyocytes were isolated from adult hearts of 16-20 week old Cas9 knock-in mice using Langendorff perfusion method. Six h after isolation, AAV6 encoding sgRNA and HDR template (1.92×10^4 viral genomes/cell) were transduced. Six days after transduction, bright-field and fluorescent images were obtained. White arrowhead indicates a rod-shaped cardiomyocyte positive for red fluorescent signal. Scale bar: 50 μm.

Figure S3

A) Analytical procedures for quantitative evaluation of DNA content in cardiomyocytes and non-cardiomyocytes using IN Cell Developer Toolbox (GE). White square of segmented images and immunostained images are enlarged as lower panels. Scale bar: 200 μm. Each italic phrase indicates the command operation defined in the software.

Nucleus Recognition

I. Select the channel with the image of the nuclei marker (Hoechst).

II. Select *Segmentation* option (greater than 65 μm^2) most appropriate for an accurate nuclear segmentation.

III. Use *Postprocessing* operations (*Erosion*, *Watershed clump breaking* and *Sieve)* to refine nuclear.

Cardiomyocyte Recognition

I. Select the channel with the image of the cardiomyocyte marker (Troponin I).

II. Select *Segmentation* option (intensity segmentation) and set an intensity threshold that identify whole cells (cell bodies).

III. Use *Postprocessing Sieve* operation (greater than 300 μm^2) to filter out debris, *Clump Breaking* operation using 'Nuclei' as 'seed'. For quantitative evaluation, we used *Border Object Removal* to omit cardiomyocytes that extend over the edge of the image.

IV. Create a linked *One to One* target set to link 'Nucleus' (primary target set) with 'Cardiomyocyte' (secondary target set), to count the number of cardiomyocytes. Set *Overlap* conditions so that more than 80% of the primary target is within the secondary target.

B) Cardiomyocytes seeded in 96-well plate were transduced with AAV6 encoding HDR components 6 h after addition of EdU, followed by continuous labeling. After 4 days of culture, cells were fixed and immunostained. Representative images of cardiomyocytes positive for tdTomato with EdU staining. Scale bar: 100 µm.

Figure S4

A) C2C12 cells were transduced with pX459 vector encoding 3xFLAG-Cas9, with or without sgRNA #2 against *Tnnt2* combined with the repair template plasmid. Two days after transfection, cells were exposed to 3 µg/mL puromycin for two days. Genomic DNA was extracted, and the targeted region was amplified by PCR. Purified PCR products were digested by *Xho*I. Arrowheads indicate the cleaved PCR products.

B) Genotyping results of ΔK210 knock-in allele in neonatal mice obtained after crossing mice carrying heterozygous ΔK210 knock-in allele and homozygous Cas9 knock-in allele. The higher PCR band indicates the

ΔK210 knock-in allele [5].

C) Neonatal cardiomyocytes isolated from Cas9 knock-in mice were transduced with AAV6 encoding LacZ or editing components shown in Fig. 4F (1.88 x 10^5 viral genomes/cell). Forty-eight hours after transduction, whole-cell lysates were extracted. Troponin T and Gapdh were detected by western blot.

D) Representative results of Sanger sequencing analysis (upper). The number of clones and their ratio in each heart sample (lower, 50 clones per sample (each sample was isolated from an individual neonatal heart; biological replicates=4)).

Figure S1

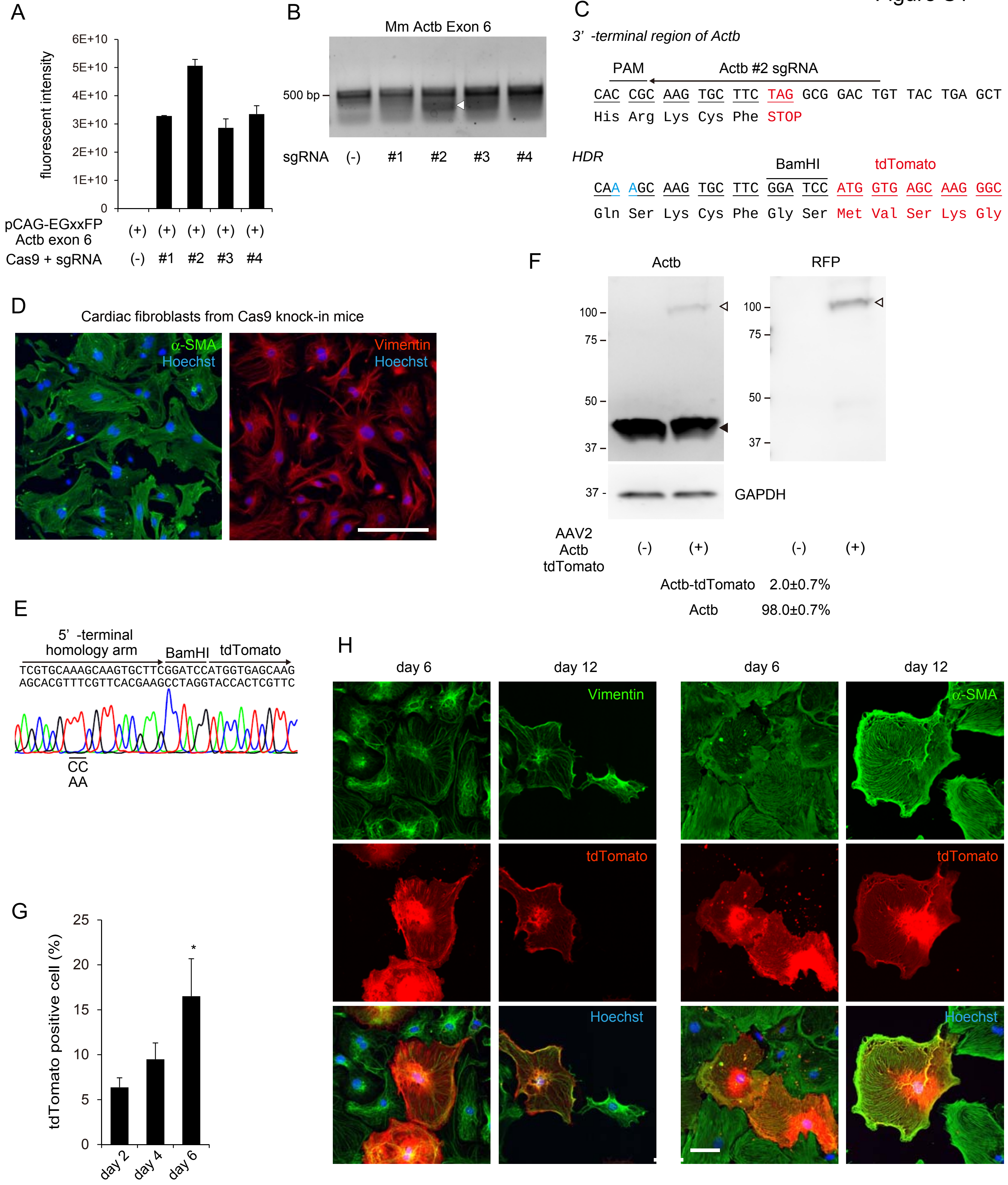

A
fluorescent intensity
pCAG-EGxxFP Actb exon 6
Cas9 + sgRNA
(-) #1 #2 #3 #4
B
Mm Actb Exon 6
500 bp
sgRNA (-) #1 #2 #3 #4
C
3' -terminal region of Actb
PAM
Actb #2 sgRNA
CAC CGC AAG TGC TTC TAG GCG GAC TGT TAC TGA GCT
His Arg Lys Cys Phe STOP
HDR
BamHI
tdTomato
CAA AGC AAG TGC TTC GGA TCC ATG GTG AGC AAG GGC
Gln Ser Lys Cys Phe Gly Ser Met Val Ser Lys Gly
D
Cardiac fibroblasts from Cas9 knock-in mice
α-SMA
Hoechst
Vimentin
Hoechst
E
5' -terminal homology arm
BamHI
tdTomato
TCGTGCAAAGCAAGTGCTTCGGATCCATGGTGAGCAAG
AGCACGTTTCGTTCACGAAGCCTAGGTACCACTCGTTC
CC
AA
F
Actb
RFP
GAPDH
AAV2 Actb tdTomato
(-) (+) (-) (+)
Actb-tdTomato 2.0±0.7%
Actb 98.0±0.7%
G
tdTomato positive cell (%)
day 2
day 4
day 6
H
day 6
day 12
Vimentin
tdTomato
Hoechst
α-SMA

Figure S2

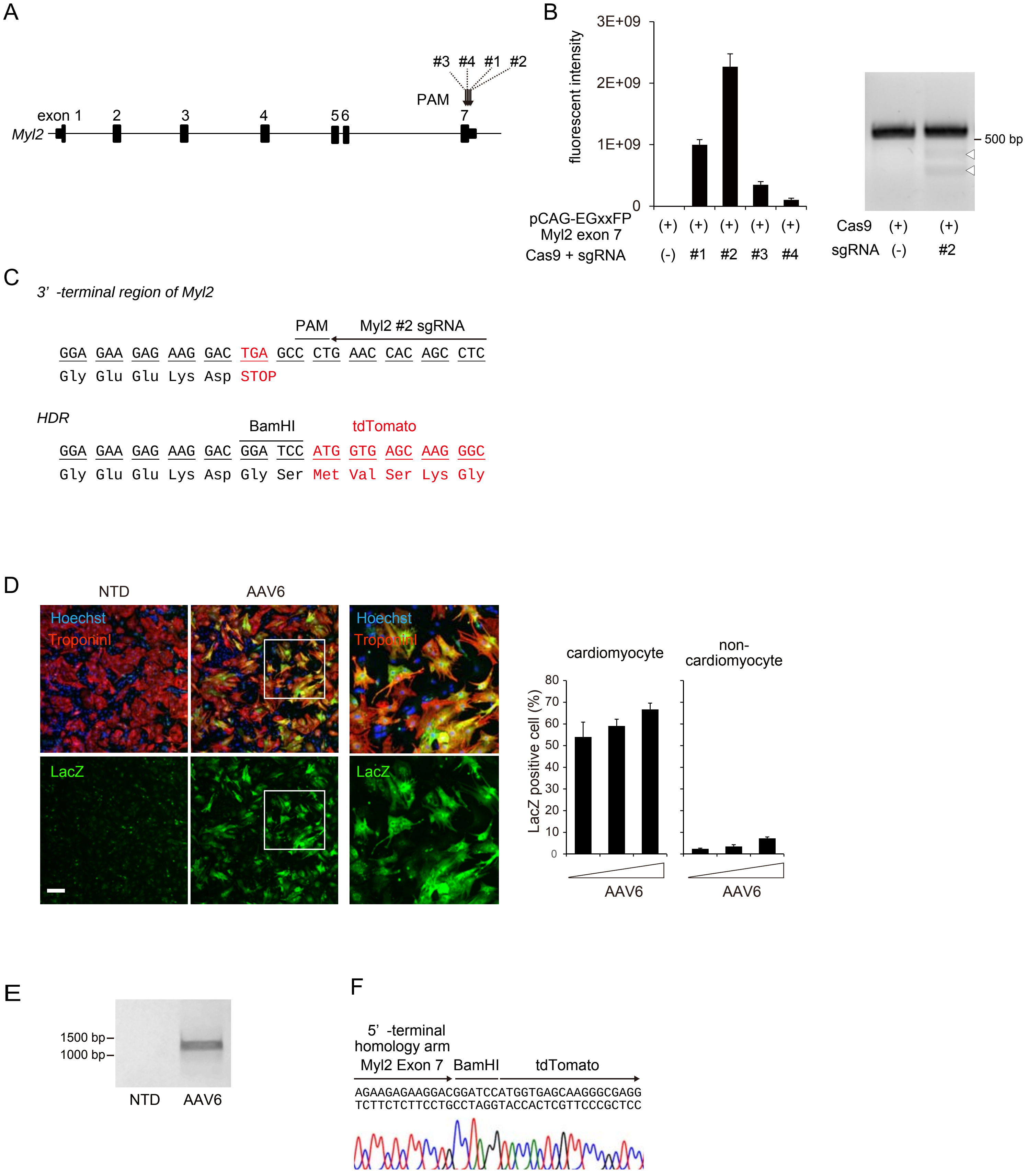
A
#3 #4 #1 #2
PAM
exon 1
2
3
4
56
7
Myl2
B
3E+09
2E+09
1E+09
0
fluorescent intensity
pCAG-EGxxFP
Myl2 exon 7
Cas9 + sgRNA
(+) (+) (+) (+) (+)
(-) #1 #2 #3 #4
500 bp
Cas9 (+) (+)
sgRNA (-) #2
C
3' -terminal region of Myl2
PAM
Myl2 #2 sgRNA
GGA GAA GAG AAG GAC TGA GCC CTG AAC CAC AGC CTC
Gly Glu Glu Lys Asp STOP
HDR
BamHI
tdTomato
GGA GAA GAG AAG GAC GGA TCC ATG GTG AGC AAG GGC
Gly Glu Glu Lys Asp Gly Ser Met Val Ser Lys Gly
D
NTD
AAV6
Hoechst
TroponinI
LacZ
cardiomyocyte
non-cardiomyocyte
LacZ positive cell (%)
80
70
60
50
40
30
20
10
0
AAV6
AAV6
E
1500 bp
1000 bp
NTD AAV6
F
5' -terminal homology arm
Myl2 Exon 7
BamHI
tdTomato
AGAAGAGAAGGACGGATCCATGGTGAGCAAGGGCGAGG
TCTTCTCTTCCTGCCTAGGTACCACTCGTTCCCGCTCC

G

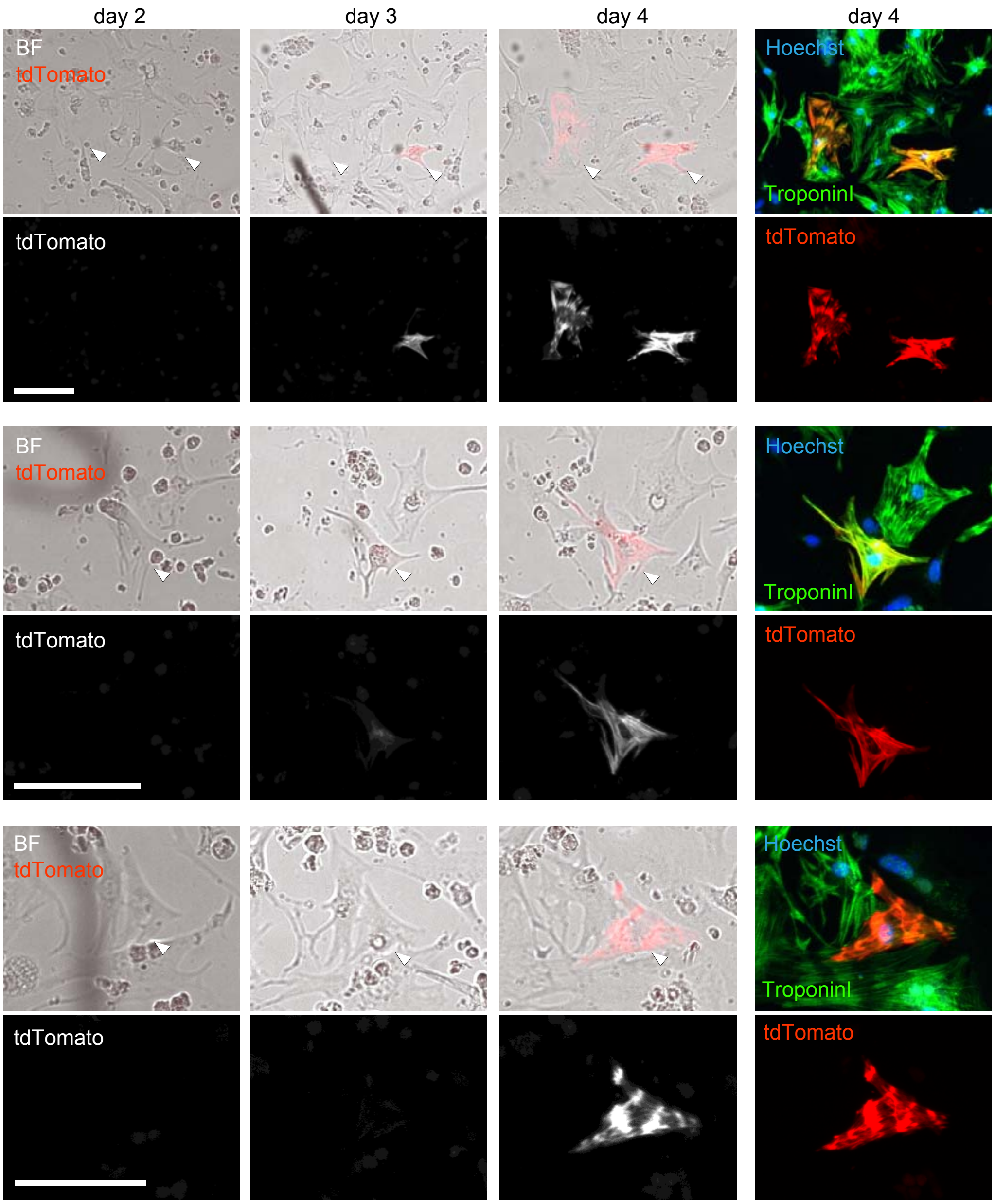
day 2
day 3
day 4
day 4
BF
tdTomato
Hoechst
TroponinI
tdTomato
tdTomato

H

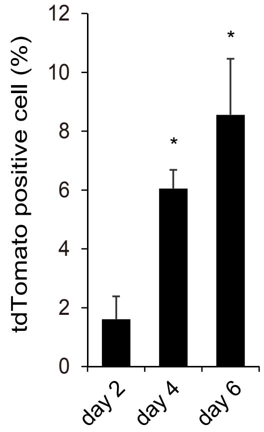
tdTomato positive cell (%)
0
2
4
6
8
10
12
*
*
day 2
day 4
day 6

J

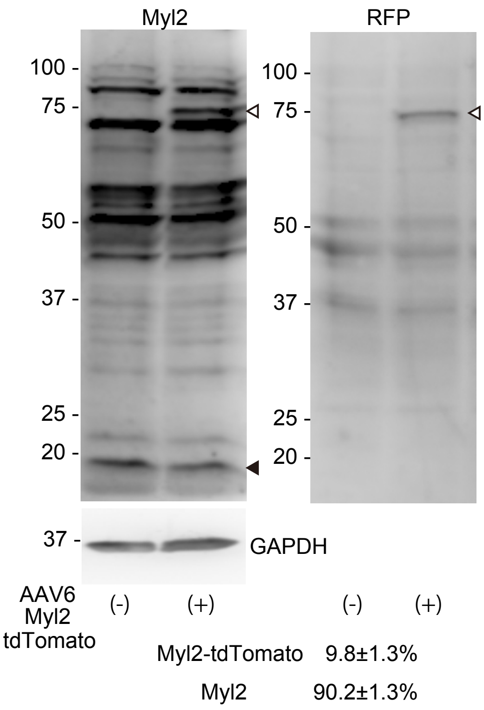
Myl2
RFP
100 -
75 -
50 -
37 -
25 -
20 -
GAPDH
AAV6
Myl2
tdTomato
(-)
(+)
Myl2-tdTomato 9.8±1.3%
Myl2 90.2±1.3%

I

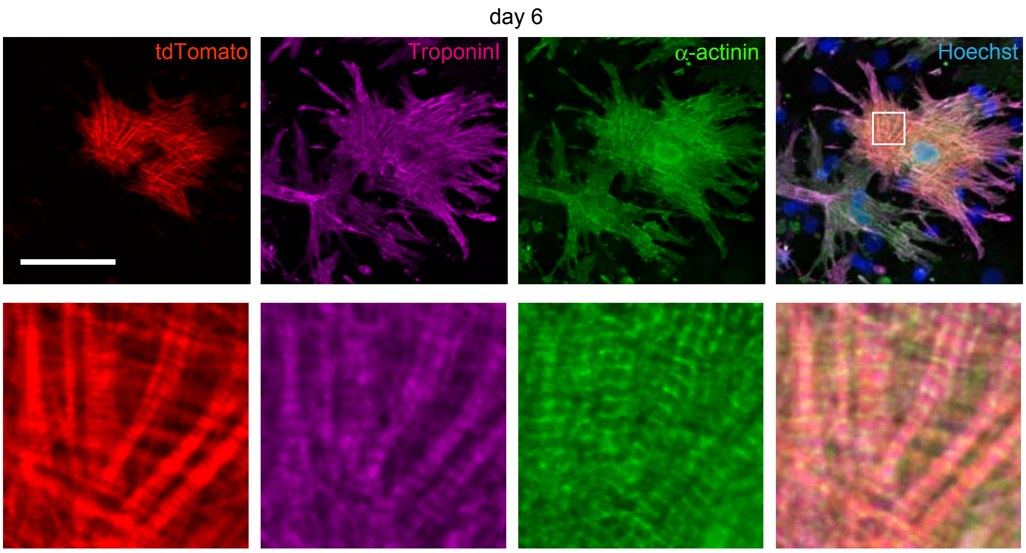
day 6
tdTomato
TroponinI
α-actinin
Hoechst

K

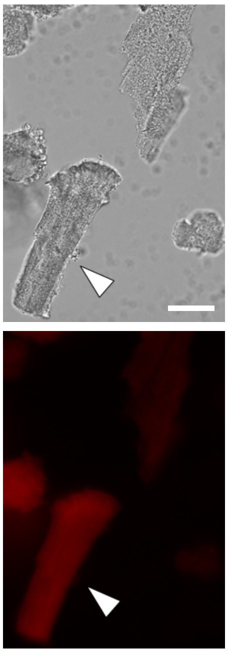

A

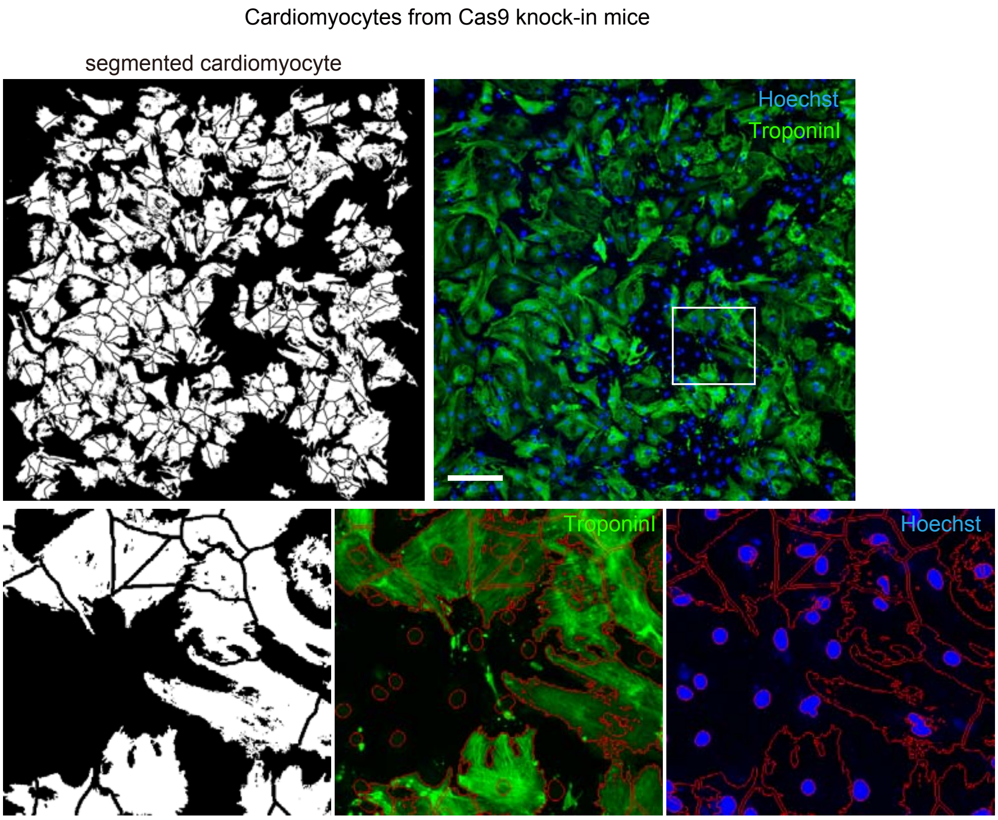
Cardiomyocytes from Cas9 knock-in mice
segmented cardiomyocyte
Hoechst
TroponinI
TroponinI
Hoechst

B

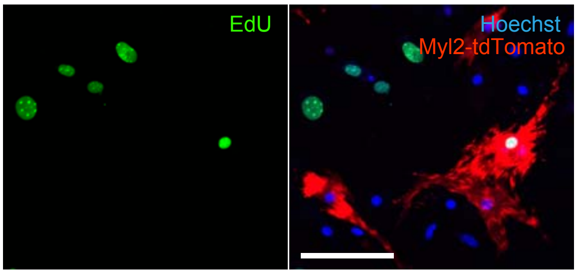
EdU
Hoechst
Myl2-tdTomato

Figure S4

A

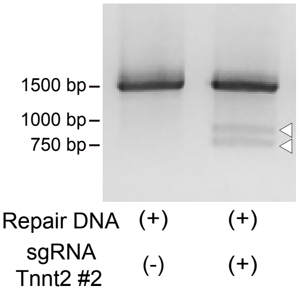

B

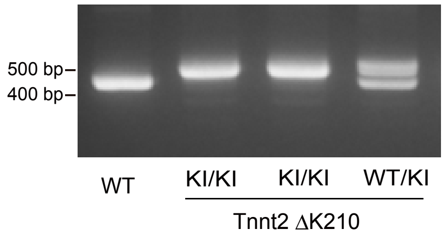

C

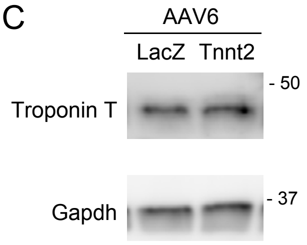

D

XhoI Exon13 ΔK210

Wildtype GGAGGAACCATCTCTAGCCAAGTGTCCCTGCTCACCATCCCCTTCAGACAGAGCGGAAGAGTGGGAAGAGACAGACAGAGAGAGAGAAGAAGAAGAAGAT

ΔK210 GGAGGAACCATCTCTAGCCAAGTGTCCCTGCTCACCATCCCCTTCAGACAGAGCGGAAGAGTGGGAAGAGACAGACAGAGAGAGAGAAGAAGAAG---AT

HDR template GGAGGAACCATCTCGAGCCAAGTGTCCCTGCTCACCATCCCCTTCAGACAGAGCGGAAGAGTGGGAAGAGACAGACAGAGAGAGAGAAGAAGAAGAAGAT

Intact GGAGGAACCATCTCTAGCCAAGTGTCCCTGCTCACCATCCCCTTCAGACAGAGCGGAAGAGTGGGAAGAGACAGACAGAGAGAGAGAAGAAGAAG---AT

NHEJ GGAGGAACCATCTCTAGCCAAGTGTCCCTGCTCACCATCCCCTTCAGACAGAGCGGAAGAGTGGGAAGAGACAGACAGAGAGAGAGAAGAAGAAG---AT

NHEJ to Exon13 GGAGGAACCATCTCTAGCCAAGTGTCCCTGCTCACCATCCCCTTCAGACAGAGCGGAAGAGTGGGAAGAGACAGACAGAGAGAGAGAAGAAGAAG---AT

HDR(PAM) GGAGGAACCATCTCTAGCCAAGTGTCCCTGCTCACCATCCCCTTCAGACAGAGCGGAAGAGTGGGAAGAGACAGACAGAGAGAGAGAAGAAGAAG---AT

HDR(ΔK210) GGAGGAACCATCTCTAGCCAAGTGTCCCTGCTCACCATCCCCTTCAGACAGAGCGGAAGAGTGGGAAGAGACAGACAGAGAGAGAGAAGAAGAAGAAGAT

HDR(XhoI) GGAGGAACCATCTCGAGCCAAGTGTCCCTGCTCACCATCCCCTTCAGACAGAGCGGAAGAGTGGGAAGAGACAGACAGAGAGAGAGAAGAAGAAGAAGAT

Exon13 sgRNA PAM

Wildtype CCTGGCAGAGAGGAGGAAGGCGCTGGCCATCGACCACCTGAATGAAGACCAACTGAGGTGGGGACAGTTGGTTGGGTGGCCCCTGGCACTCTTCCTGAGT

ΔK210 CCTGGCAGAGAGGAGGAAGGCGCTGGCCATCGACCACCTGAATGAAGACCAACTGAGGTGGGGACAGTTGGTTGGGTGGCCCCTGGCACTCTTCCTGAGT

HDR template CCTGGCAGAGAGGAGGAAGGCGCTGGCCATCGACCACCTGAATGAAGACCAACTGAGGTGGGGACAGTTGGTTGGGTGGCCCCAAACACTCTTCCTGAGT

Intact CCTGGCAGAGAGGAGGAAGGCGCTGGCCATCGACCACCTGAATGAAGACCAACTGAGGTGGGGACAGTTGGTTGGGTGGCCCCTGGCACTCTTCCTGAGT

NHEJ CCTGGCAGAGAGGAGGAAGGCGCTGGCCATCGACCACCTGAATGAAGACCAACTGAGGTGGGGACAGTTGGTT-------CCCTGGCACTCTTCCTGAGT

NHEJ to Exon13 CCTGGCAGAGAGGAGGAAGGCGCTGGCCATCGACCAC--------------------------<112bp>--------------------------

HDR(PAM) CCTGGCAGAGAGGAGGAAGGCGCTGGCCATCGACCACCTGAATGAAGACCAACTGAGGTGGGGACAGTTGGTTGGGTGGCCCCAAACACTCTTCCTGAGT

HDR(ΔK210) CCTGGCAGAGAGGAGGAAGGCGCTGGCCATCGACCACCTGAATGAAGACCAACTGAGGTGGGGACAGTTGGTTGGGTGGCCCCAAACACTCTTCCTGAGT

HDR(XhoI) CCTGGCAGAGAGGAGGAAGGCGCTGGCCATCGACCACCTGAATGAAGACCAACTGAGGTGGGGACAGTTGGTTGGGTGGCCCCAAACACTCTTCCTGAGT

	heart #1		heart #2		heart #3		heart #4	
Intact	23	(46%)	13	(26%)	31	(62%)	13	(26%)
NHEJ	17	(34%)	23	(46%)	10	(20%)	28	(56%)
NHEJ to Exon13	1	(2%)	1	(2%)	2	(4%)	3	(6%)
HDR(PAM)	2	(4%)	3	(6%)	1	(2%)	4	(8%)
HDR(ΔK210＋XhoI)	7	(14%)	10	(20%)	6	(12%)	2	(4%)
total clone	50		50		50		50	
